# RELAY, ramucirumab plus erlotinib versus placebo plus erlotinib in untreated *EGFR*-mutated metastatic non-small cell lung cancer: exposure–response relationship

**DOI:** 10.1007/s00280-022-04447-x

**Published:** 2022-07-16

**Authors:** Kazuhiko Nakagawa, Edward B. Garon, Ling Gao, Sophie Callies, Annamaria Zimmermann, Richard Walgren, Carla Visseren-Grul, Martin Reck

**Affiliations:** 1 Department of Medical Oncology, Faculty of Medicine, Kindai University, Osakasayama City, 377-2, Ohno-higashi, Osaka, 589-8511 Japan; 2 David Geffen School of Medicine at University of California Los Angeles, Translational Research in Oncology US Network, Los Angeles, CA USA; 3 Eli Lilly and Company, Bridgewater, USA; 4 Eli Lilly and Company, Paris, France; 5 Eli Lilly and Company, Indianapolis, IN USA; 6 Eli Lilly Netherlands, Utrecht, The Netherlands; 7 LungenClinic, Airway Research Center North, German Center for Lung Research, Grosshansdorf, Germany; 8 Kindai University Faculty of Medicine, Osaka, Japan

**Keywords:** Non-small cell lung cancer, Ramucirumab, Exposure–response, Pharmacokinetics

## Abstract

**Purpose:**

In RELAY, ramucirumab plus erlotinib (RAM + ERL) improved progression-free survival (PFS) in patients with untreated, metastatic, EGFR-mutated, non-small cell lung cancer (NSCLC). Here, we present the exposure–response relationship of RAM from RELAY.

**Methods:**

Patients received ERL (150 mg/day) with either RAM (10 mg/kg) or placebo (PBO + ERL) every 2 weeks (Q2W). A population pharmacokinetic model predicted RAM minimum concentration after first dose (*C*_min,1_), and at steady state (*C*_min,*ss*_), which were used to evaluate correlation between RAM exposure and efficacy and safety. The Kaplan–Meier method and Cox regression analyses were utilized to evaluate exposure–efficacy by *C*_min,1_ quartile. Exposure–safety was evaluated by assessing incidence rates for safety parameters by *C*_min,*ss*_ quartile, with ordered categorical analysis used for ALT/AST only.

**Results:**

Analyses included 216 patients treated with RAM + ERL and 225 patients treated with PBO + ERL. Adjusting for significant baseline covariates, no exposure–efficacy relationship was identified in RELAY: PFS hazard ratio (mean, 95% confidence intervals) for the *C*_min,1_ quartiles were 0.67 (0.45–0.99), 0.77 (0.53–1.12), 0.57 (0.38–0.84), and 0.50 (0.33–0.76). No apparent exposure–safety relationship was observed for selected safety endpoints, including Grade ≥ 3 hypertension, diarrhea, and dermatitis acneiform, and any grade hypertension, any grade and Grade ≥ 3 proteinuria, and any grade ALT/AST increased within liver failure/liver injury.

**Conclusions:**

No association was observed between RAM exposure and response, suggesting that the RELAY regimen of RAM 10 mg/kg Q2W with ERL is an optimized, efficacious, and safe first-line treatment for patients with untreated, metastatic, EGFR-mutated NSCLC.

*Trial registration:* ClinicalTrials.gov, NCT02411448.

**Supplementary Information:**

The online version contains supplementary material available at 10.1007/s00280-022-04447-x.

## Introduction

Non-small cell lung cancer (NSCLC) is the most common subtype of lung cancer, accounting for approximately 85% of cases. As NSCLC is a heterogeneous disease, a personalized approach to treatment based on the mutation profile of the tumor has been proven to be the most effective strategy [1]. *Epidermal growth factor receptor* (*EGFR*) mutations are among the most prevalent genetic aberrations in NSCLC, detected in approximately 50% of Asian patients, and 10–15% of Western patients [2]. Mutations of the *EGFR* gene lead to constitutive activation of pathways involved in cell proliferation and division through aberrant activity of the encoded receptor tyrosine kinase. The most common *EGFR* mutations are an exon 19 deletion and an L858R mutation on exon 21, both of which are sensitive to *EGFR* tyrosine kinase inhibitors (EGFR TKIs) [1]. Due to the clinical benefits of EGFR TKIs in *EGFR-*mutant NSCLC, these agents are widely used as first-line standard of care [3, 4]. However, despite the efficacy of EGFR TKIs, eventually all patients develop treatment resistance [1, 2, 5]. Thus, novel treatment strategies that delay or prevent acquired resistance and enhance efficacy in *EGFR*-mutant NSCLC are required. To this end, combining therapies with complementary anticancer mechanisms is an attractive method to improve clinical outcome.

The vascular endothelial growth factor (VEGF) pathway is implicated in many cancers including NSCLC [6]. Through overexpression of the VEGF family of growth factors, tumor cells can trigger the formation of new vasculature and increase oxygen and blood supply in the tumor microenvironment. Ramucirumab (Cyramza^®^), a recombinant human monoclonal antibody (mAb), selectively binds vascular endothelial growth factor receptor 2 (VEGFR2). By blocking the interaction between the VEGF ligand and its receptor, ramucirumab prevents activation of the VEGF signaling cascade, ultimately inhibiting angiogenesis. Not only have *EGFR*-mutant tumors demonstrated increased VEGF dependence compared to *EGFR*-wildtype tumors, but VEGF inhibitors have also demonstrated the ability to enhance antitumor activity in *EGFR* T790M positive cancer cells [7–10]. Accordingly, as both molecular signaling pathways are synergistically involved in tumor growth, combining epidermal growth factor (EGF) inhibition with a VEGF inhibitor such as ramucirumab is a rational treatment strategy in *EGFR*-mutant NSCLC. Indeed, clinical studies have demonstrated the ability of dual EGFR/VEGF pathway inhibition to improve clinical outcomes [11–13].

During the clinical development of anticancer agents, it is imperative to establish a treatment regimen that minimizes toxicity and maximizes efficacy in all patients [14, 15]. While doses administered may be efficacious in some patients, it may not provide the optimal drug concentration across the entire patient cohort. Therefore, exposure–response (E–R) analyses are routinely conducted to support the selection of a dosing regimen, whereby population pharmacokinetics (PopPK) are modeled and simulated to evaluate the relationship between drug exposure levels, efficacy, and safety [14].

Ramucirumab is administered at different doses and dosing schedules depending on its indication. The REGARD and RAINBOW trials demonstrated the efficacy of ramucirumab (8 mg/kg) once every 2 weeks (Q2W) over placebo in gastric or gastroesophageal junction cancer [16, 17]. Tabernero et al. conducted an E–R analysis to assess if increased exposure could maximize the therapeutic benefit [18]. The exposure–efficacy analysis, which looked at progression-free survival (PFS) and overall survival (OS) by predicted minimum concentration of ramucirumab at steady-state (*C*_min,*ss*_) quartile demonstrated a positive relationship between efficacy and exposure in gastric malignancies [18]. Importantly, a similar positive exposure–efficacy relationship was observed in patients with NSCLC treated with ramucirumab in combination with docetaxel in the REVEL trial [19]. In REVEL, a randomized, double-blind Phase 3 trial, patients with previously treated Stage IV NSCLC received either ramucirumab (10 mg/kg) or placebo once every 3 weeks (Q3W) plus docetaxel [20]. Smit et al. conducted an E–R analysis of REVEL data to evaluate the relationship between ramucirumab exposure and PFS, OS, and treatment emergent adverse events (TEAEs) [19]. Evaluation of Kaplan–Meier-estimated PFS and OS indicated an E–R relationship with the Q3W dosing schedule, with higher ramucirumab exposure being associated with improved clinical outcomes and increased toxicities.

The results of these E–R analyses suggest that patients with lower serum ramucirumab concentrations respond less optimally compared to patients with the highest exposure (reaching a predicted minimum concentration after first dose [*C*_min,1_] > 50 µg/mL) [19]. Thus, a treatment regimen that generates optimized drug concentrations across the full patient population could increase the possibility of achieving consistent therapeutic benefit.

With the goal of optimizing ramucirumab serum concentration, RELAY implemented a 10 mg/kg Q2W dosing regimen [13]. This regimen was predicted to lead to higher average and minimum concentration as compared to the regimen used in REVEL (10 mg/kg Q3W), whereas the impact on Cmax was predicted to be minimal. Here we present the E–R relationship of ramucirumab using patient data from the RELAY trial [21]. The objective was to determine if Q2W dosing achieves optimal serum concentrations across the patient cohort, increasing the chance of consistent therapeutic effect.

## Methods

### Study design

RELAY is a global, randomized, double-blind placebo-controlled, Phase 3 study that included treatment-naive patients with metastatic NSCLC and an *EGFR* Exon 19del or Exon 21_L858R mutation. Patients were randomized (1:1) to receive erlotinib (150 mg/day) as an oral dose with either ramucirumab (10 mg/kg) (RAM + ERL) or placebo (PBO + ERL) as an intravenous infusion over approximately one-hour Q2W. Dose modifications were permitted to allow recovery from toxic effects. Ramucirumab could be delayed for up to 42 days and three steps of dose reduction were permitted (to 8 mg/kg, 6 mg/kg, and 5 mg/kg). Erlotinib could be delayed for up to 3 weeks and two steps of dose reduction were permitted (to 100 mg/day and 50 mg/day).

The primary endpoint, PFS, was reported previously [13]. Key secondary endpoints included analysis of ramucirumab PK parameters, safety, and efficacy. Samples for PK analysis were collected from patients at baseline, pre-infusion at cycles 1, 2, 4, 7, 14, one-hour post-infusion at cycles 1 and 14, and at the 30-day post study treatment discontinuation follow-up visit. Serum ramucirumab concentration was determined using a validated enzyme-linked immunosorbent assay [22]. The protocol and amendments were approved by the ethics committees of all participating centres and all patients provided written informed consent before study entry. The trial was conducted according to the Declaration of Helsinki, the International Conference on Harmonisation guidelines for good clinical practice, and applicable local regulations. Detailed methods of this trial have been reported previously [13].

### PopPK model development—data and analysis method

Population pharmacokinetic (PopPK) modeling is widely used in drug development to describe a drug concentration profile over time and define inter- and intra-patient variability. The ramucirumab PopPK model is based on a large ramucirumab PK data set comprising of 12,797 PK observations in 2,820 patients and includes data from 18 Phase 1 to 3 studies in patients with colorectal, hepatic, gastric, non-small cell lung, urothelial, breast cancer and other solid tumor types. The ramucirumab dose levels range from 6 to 12 mg/kg (given as 6 mg/kg QW, 8 mg/kg Q2W, 10 mg/kg Q2W, 12 mg/kg Q2W, 10 mg/kg Q3W and 8 mg/kg on D1, D8 of Q3W cycle).The PopPK model was developed as previously described [23], using a non-linear mixed-effects modeling approach (NONMEM version 7.4.2).

### PopPK model application

Using the PopPK model, the predicted minimum concentrations after first-dose administration (*C*_min,1_) and at steady state (*C*_min,*ss*_) were determined for ramucirumab-treated patients. Analyses were conducted in accordance with the Food and Drug Administration (FDA) Guidance for Industry on Population Pharmacokinetics [24].

### Exposure efficacy analysis

Both univariate and multivariate Cox regression analyses were performed to evaluate the relationship between minimum concentration after the first dose (*C*_min,1_) and PFS. Though no preclinical study was conducted to demonstrate *C*_min,1_ was the driver for efficacy, preclinical data showed tumor shrinkage was observed in a mice xenograft model with steady state *C*_min_ ramucirumab concentration greater than a threshold limit of approximately 20 ug/mL [25]. Consequently *C*_min,1_ was used instead of *C*_min,*ss*_ as the objective was to achieve the target efficacious concentration as early as possible. Thus, *C*_min,1_ was selected for the E–R analysis. This approach is supported by an E–R analysis of the monoclonal antibody, nivolumab, conducted by the FDA [26, 27] and was used for the ramucirumab E–R analysis in NSCLC REVEL study [19]. Only patients who had available concentration data were included in the analyses. In the univariate analyses, the exposure measure of interest was included as the only covariate in the model and was treated as a continuous variable. In the multivariate Cox regression analyses, separate models were fitted using exposure measures as either continuous or categorical variables (quartile groups). Using a Cox proportional hazard model, hazard ratios (HR) were estimated and were adjusted for significant baseline covariates. Factors with potential prognostic significance were identified using a stepwise Cox regression with an entry *p* value of < 0.05 and an exit *p* value of ≥ 0.1. These factors included *EGFR* mutation type, gender, region, *EGFR* testing method, age, smoking history, performance status, initial stage at diagnosis, and liver metastasis.

To evaluate the exposure–efficacy relationship compared with the PBO + ERL treatment arm, patients in the RAM + ERL treatment arm were defined by *C*_min,1_ exposure quartiles (*Q*): Q1 *C*_min,1_ 4.13–31.6 μg/mL (< 25%); Q2, *C*_min,1_ 31.8–37.6 μg/mL (25–< 50%); Q3, *C*_min,1_ 37.7–42.9 μg/mL (50–< 75%); Q4, *C*_min,1_ 43.0–59.9 μg/mL (> 75%). The Kaplan–Meier method was used to evaluate PFS for each of the individual quartiles versus the PBO + ERL treatment arm. The HR for each quartile versus the control arm was estimated using a Cox proportional hazards model. All HRs for the multivariable analyses were adjusted for the significant baseline covariates.

Additional case-matched control analyses for PFS were explored to adjust for potential imbalances in significant prognostic factors between the treatments within each exposure quartile group as previously explained [28]. In these analyses, the case groups were the exposure quartiles of predicted *C*_min,1_ in the RAM + ERL treatment arm. For every patient in each case group, a matched control patient was selected from all patients receiving PBO + ERL through a matching scheme based on the imbalance in baseline characteristics and important prognostic factors identified in the stepwise Cox regression analyses. The Mahalanobis metric matching (with a caliper size of 1/4 standard deviation of the logit score) was used [29]. The balance of the selected significant patient factors between the two treatments was assessed in each case–control group, before and after matching, using Fisher’s exact test or *t* test. Note, missing values in any of the matching factors excluded the patients from the matched analysis. Kaplan–Meier survival analysis and Cox models were performed to compare the 2 treatments in each of the matched case–control groups. The statistical analyses were conducted using SAS software (SAS Enterprise Guide 7.15).

### *Exposure*–*safety analysis*

The safety endpoints selected for exposure–safety analysis were the most common Grade ≥ 3 TEAEs from RELAY, occurring in at least 5% of patients in the ramucirumab plus erlotinib treatment arm and with a higher incidence (≥ 2%) than the placebo plus erlotinib treatment arm. These TEAEs were hypertension, diarrhea, and dermatitis acneiform and were graded per the National Cancer Institute Common Terminology Criteria for Adverse Events version 4.0. In addition, selected adverse events of special interest (AESI), including hypertension (any grade), proteinuria (any grade and Grade ≥ 3), and liver failure/liver injury, were also evaluated. Ordered categorical models were developed to evaluate the relationship between the predicted ramucirumab concentrations (*C*_min,1_ and *C*_min,*ss*_) and TEAEs.

## Results

### Exposure–response population

The RELAY intent-to-treat (ITT) population included 449 randomized patients, 224 patients randomized to receive RAM + ERL, and 225 patients randomized to receive PBO + ERL. Among the 224 patients randomized to the RAM + ERL treatment arm, three patients did not receive study treatment, and a further 5 patients had no evaluable PK data. Consequently, data from 216 patients from the RAM + ERL treatment arm and 225 patients from the PBO + ERL treatment arm were included in the exposure–response analysis. The baseline demographics and disease characteristics were well balanced between the treatment groups in the exposure response population and consistent with the ITT population, suggesting that the exposure–response population is reflective of the ITT population enrolled in RELAY (Table 1).

**Table 1:** RELAY Baseline patient demographics and disease characteristics by *C*_min,1_ quartile

	ITT population		Exposure–response population				
	PBO + ERL	RAM + ERL	PBO + ERL	RAM + ERL^a^			
				*C*_min,1_			
				RAM Q1	RAM Q2	RAM Q3	RAM Q4
	*N* = 225	*N* = 224	*N* = 225	*N* = 54	*N* = 54	*N* = 54	*N* = 54
*Sex, n (%)*							
Male	83 (37)	83 (37)	83 (37)	21 (39)	23 (43)	25 (46)	12 (22)
Female	142 (63)	141 (63)	142 (63)	33 (61)	31 (57)	29 (54)	42 (78)
*Age, years*							
Mean (SD)	62.9 (10.6)	63.7 (10.2)	62.9 (10.6)	63.0 (11.8)	63.6 (10.4)	64.1 (10.3)	63.9 (8.8)
Median (min–max)	64 (23–89)	65 (27–86)	64 (23–89)	65.0 (27–84)	64.5 (41–86)	65.0 (41–83)	65.0 (43–83)
*Race, n (%)*							
Asian	174 (77)	172 (77)	174 (77)	47 (87)	38 (70)	43 (80)	39 (72)
Caucasian	48 (21)	52 (23)	48 (21)	7 (13)	16 (30)	11 (20)	15 (28)
Other	3 (1)	0 (0)	3 (1)	0 (0)	0 (0)	0 (0)	0 (0)
*Geographic region*^*b*^*, n (%)*							
East Asia	170 (76)	166 (74)	170 (76)	43 (80)	37 (69)	43 (80)	38 (70)
Other	55 (24)	58 (26)	55 (24)	11 (20)	17 (32)	11 (20)	16 (30)
*ECOG PS, n (%)*							
0	119 (53)	116 (52)	119 (53)	23 (43)	27 (50)	31 (57)	30 (56)
1	106 (47)	108 (48)	106 (47)	31 (57)	27 (50)	23 (43)	24 (44)
*Smoking history, n (%)*							
Ever	73 (32)	64 (29)	73 (32)	14 (26)	17 (32)	15 (28)	16 (30)
Never	139 (62)	134 (60)	139 (62)	30 (56)	30 (56)	33 (61)	35 (65)
Unknown	13 (6)	26 (12)	13 (6)	10 (19)	7 (13)	6 (11)	3 (6)
*Histological diagnosis, n (%)*							
Adenocarcinoma	218 (97)	215 (96)	218 (97)	51 (94)	51 (94)	53 (98)	53 (98)
NSCLC-NOS	7 (3)	9 (4)	7 (3)	3 (6)	3 (6)	1 (2)	1 (2)
*EGFR mutation type*^*c*^*, n (%)*							
Exon 19	120 (53)	123 (55)	120 (53)	33 (61)	26 (48)	33 (61)	29 (54)
Exon 21	105 (47)	99 (44)	105 (47)	21 (39)	28 (52)	19 (35)	25 (46)
Other	0 (0)	1 (< 1)	0 (0)	0 (0)	0 (0)	1 (2)	0 (0)
Missing	0 (0)	1 (< 1)	0 (0)	0 (0)	0 (0)	1 (2)	0 (0)
Liver metastasis, *n* (%)	24 (11)	21 (9)	24 (11)	6 (11)	7 (13)	6 (11)	2 (4)
*Body weight (kg)*							
Mean (CV%) (min–max)	60.8 (22) (35.8–117.0)	60.7 (20) (33.9–94.0)	60.8 (22) (35.8–117.0)	55.8 (21) (33.4–83.3)	57.5 (14) (39.1–73.4)	64.0 (20) (40.5–94.0)	64.9 (19) (46.0–92.0)

^a^PK data were not available for 8 patients, so analyses were conducted in 216 patients in the ramucirumab plus erlotinib group

^b^East Asia includes South Korea, Hong Kong, Japan, and Taiwan, and Other includes Canada, France, Germany, Italy, Romania, Spain, Turkey, USA, and UK

^c^As recorded in the case report form; predicted *C*_min,1_ exposure quartiles: RAM Q1 *C*_min,1_ 4.13–31.6 μg/mL (< 25%); RAM Q2, *C*_min,1_ 31.8–37.6 μg/mL (25–< 50%); RAM Q3, *C*_min,1_ 37.7–42.9 μg/mL (50–< 75%); RAM Q4, *C*_min,1_ 43.0–59.9 μg/mL (≥ 75%); *C*_min,1_, minimum concentration after first dose; *N*, number of patients, ECOG PS, Eastern Cooperative Oncology Group Performance Status, EGFR, epidermal growth factor receptor, CV, coefficient of variation, NSCLC-NOS, non-small cell lung cancer not otherwise specified, ITT, Intent to treat population; Q, quartile; RAM, ramucirumab

### Pharmacokinetic results

The ramucirumab PK data are well described by a two compartmental disposition PK model with time varying clearance. The model was parameterized in terms of drug clearance CL, central volume of distribution (*V*_1_), peripheral volume of distribution (*V*_2_), and inter-compartmental clearance (*Q*). Exponential inter-patient variability terms were included for CL, *V*_1_, and *V*_2_. The model included weight effect on clearance and central volume of distribution [22]. The model also includes a non-linear relationship (sigmoidal Emax relationship) to describe the decrease over time of ramucirumab clearance with an additive inter-patient variability on Emax (maximal change in CL). This non-linear clearance over time phenomenon was also described for other monoclonal antibodies such as nivolumab [27]. Covariance was estimated between CL and *V*_1_ and between CL and *E*_max_. The residual variability was characterized using combined proportional and additive components.

Objective function mapping and VPC showed that the parameters were well estimated, and the model accurately described the data. Further, ramucirumab PK was similar between East-Asian and non-East-Asian (mostly Caucasian) patients (Online Resource 1).

**Fig. 1:**
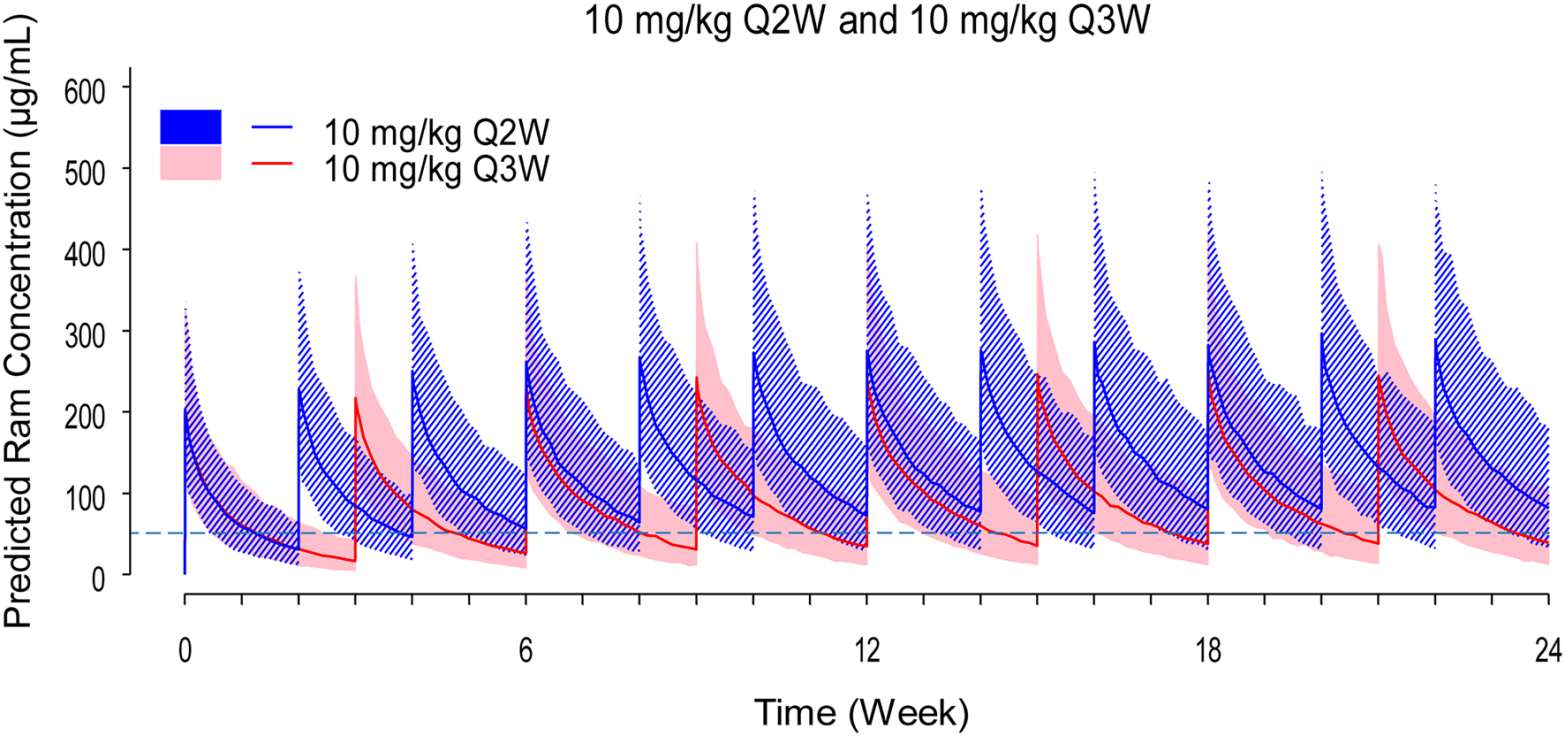
RELAY predicted ramucirumab concentration–time profiles following 10 mg/kg Q2W in RELAY compared with 10 mg/kg Q3W in REVEL. Shaded regions represent the 5th and 95th percentile ramucirumab concentrations calculated from 500 simulation iterations. The dashed horizontal line indicates ramucirumab *C*_min,*ss*_ of 50 ug/mL. Q2W, on Day 1 of each 2-week cycle; Q3W, on Day 1 of each 3-week cycle

Simulations were performed using the final model parameter estimates to compare ramucirumab concentration versus time profiles for a typical patient on the 10 mg/kg Q2W regimen with the approved 10 mg/kg once every three weeks (Q3W) dosing regimen in 2L NSCLC. This comparative analysis was conducted using patient PK data from the REVEL study. Dose amounts were calculated by sampling from the body weight distribution in the PopPK patient population. Based on the simulations visually depicted in Fig. 1, ramucirumab steady-state concentration levels were higher following 10 mg/kg Q2W compared with 10 mg/kg Q3W dosing. Although the Q2W regimen produced greater overall exposure, there was only a slight increase in maximum serum concentration (*C*_max_) in comparison to the 3-week regimen.

Table 2 depicts the ramucirumab concentrations at steady state observed in this study and reported in the REVEL study. In REVEL, 10 mg/kg Q3W dosing resulted in a drug concentration range of 54.9–117 µg/mL in the 4th quartile. Comparatively, the 10 mg/kg Q2W dosing regimen followed in RELAY delivered, in ≥ 90% of patients, *C*_min,*ss*_ drug concentrations equivalent to the upper 4th quartile levels achieved in REVEL, i.e. above the target 50 µg/mL.

**Table 2:** Ramucirumab steady-state concentration levels following 10 mg/kg Q2W compared with 10 mg/kg Q3W dosing

	Ramucirumab *C*_min,*ss*_ (µg/mL)
*REVEL*	
Ramucirumab Q3W *C*_min,*ss*_ 4th upper quartile	54.9–117
*RELAY*	
Ramucirumab Q2W *C*_min,*ss*_ geomean (CV%)	85.7 (32)
*Range*	36–197
RELAY percentiles	
5th	48.6
10th	58.0
20th	65.6

*C*_min,*ss*_, minimum concentration at steady-state; *Geomean*, geometric mean; *CV*, coefficient of variation; *Q2W*, on Day 1 of each 2-week cycle; *Q3W*, on Day 1 of each 3-week cycle

Observed exposure parameters for erlotinib are shown in Online Resource 2. Erlotinib PK was assessed in a limited number of patients (*n* < 15) as monotherapy in the placebo arm, and in combination with ramucirumab in the experimental arm. Distribution of erlotinib exposure parameters in plasma were generally similar between the treatment arms. The ratios of geometric least squares means and 90% confidence intervals (CIs) at 1.23 (90% CI 1.02–1.50) for AUC_24_ and 1.14 (90% CI 0.97–1.34) for *C*_max_, indicate that coadministration with ramucirumab is unlikely to affect erlotinib PK.

### *Exposure*–*efficacy analyses*

**Fig. 2:**
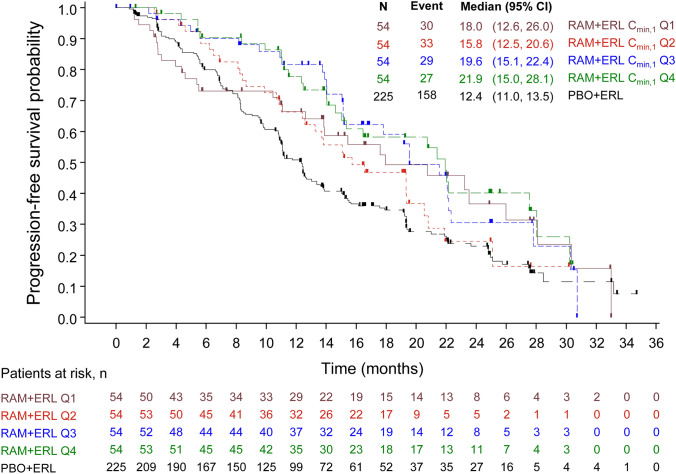
RELAY Progression free survival predicted by *C*_min,1_ quartile. Predicted *C*_min,1_ exposure quartiles: RAM Q1 *C*_min,1_ 4.13–31.6 μg/mL (< 25%); RAM Q2, *C*_min,1_ 31.8–37.6 μg/mL (25–< 50%); RAM Q3, *C*_min,1_ 37.7–42.9 μg/mL (50–< 75%); RAM Q4, *C*_min,1_ 43.0–59.9 μg/mL (≥ 75%). *C*_min,1_, minimum concentration after first dose; Q, quartile; RAM + ERL, ramucirumab plus erlotinib; PBO + ERL, placebo plus erlotinib; N, number of patients in group

A univariate Cox regression analysis of the efficacy data from the ramucirumab arm with *C*_min,1_ as the continuous covariate showed that the association between *C*_min,1_ and PFS was not statistically significant [HR 0.841 (95% confidence interval: 0.594, 1.192); *p* = 0.3309]. After adjusting for the baseline prognostic factor of performance status (ECOG PS 0–1), the relationship between ramucirumab exposure (*C*_min,1_) and PFS was still not statistically significant (*p* = 0.2971). Ramucirumab exposure was also evaluated as a categorical covariate for comparisons with the PBO + ERL group. Kaplan–Meier plots of PFS by *C*_min,1_ quartile demonstrated apparent separation between the PBO + ERL arm and each RAM + ERL quartile (Fig. 2). The median PFS was 18.0, 15.8, 19.6, 21.9, and 12.4 months for Q1, Q2, Q3, Q4, and placebo, respectively. The median PFS values from all exposure quartiles were longer than that of PBO + ERL arm, but no clear exposure–response relationship was observed within the exposure range following 10 mg/kg Q2W in the study. Results from multivariate cox regression analysis, shown in Table 3, demonstrated that all four RAM + ERL quartiles showed strong treatment effect for PFS compared with the PBO + ERL arm, with HRs ranging from 0.504 to 0.769. With significant overlap between the confidence intervals of the lowest HR [mean 0.504 (95% CI 0.334–0.759)], observed in the Q4 group, and the highest HR [mean 0.769 and (95% CI 0.528–1.12)], observed in the Q2 group, no apparent E–R trend was observed.

**Table 3:** RELAY Multivariate Cox regression analysis of progression-free survival by *C*_min,1_ quartile

	PBO + ERL N^a^	RAM + ERL N^a^	Hazard-ratio (95% CI)
RELAY ITT population^b^			
RAM + ERL *C*_min,1_ Q1 versus PBO + ERL	225	54	0.671 (0.452, 0.994)
RAM + ERL *C*_min,1_ Q2 versus PBO + ERL	225	54	0.769 (0.528, 1.120)
RAM + ERL *C*_min,1_ Q3 versus PBO + ERL	225	54	0.566 (0.381, 0.843)
RAM + ERL *C*_min,1_ Q4 versus PBO + ERL	225	54	0.504 (0.334, 0.759)

^a^Patients with missing baseline covariate factors were omitted from the analysis

^b^Adjusted for ECOG PS (0 vs. 1). Predicted *C*_min,1_ exposure quartiles: RAM Q1 *C*_min,1_ 4.13–31.6 μg/mL (< 25%); RAM Q2, *C*_min,1_ 31.8–37.6 μg/mL (25–< 50%); RAM Q3, *C*_min,1_ 37.7–42.9 μg/mL (50–< 75%); RAM Q4, *C*_min,1_ 43.0–59.9 μg/mL (≥ 75%). *C*_min,1_, minimum concentration after first dose; *ITT*, intent to treat; CI, confidence interval; *N*, number of patients in group; *Q*, quartile; RAM + ERL, ramucirumab plus erlotinib; PBO + ERL, placebo plus erlotinib

We adjusted for the potential impact of imbalances and important prognostic factors between the treatment arms within each exposure group using a matched case–control analysis for PFS. Two matching factors with prognostic significance associated with PFS were identified and adjusted for age (< 65 vs. ≥ 65), and ECOG PS at baseline (0 vs. 1). Matching was performed separately within each *C*_min,1_ exposure quartile in the RAM + ERL arm (Online Resource 3). Overall, the results from case-matched control analysis were similar to those observed in the multivariate cox regression analysis when compared with the entire PBO + ERL group (data not shown).

### *Exposure*–*safety analyses*

Observed incidences of Grade ≥ 3 hypertension, diarrhea, proteinuria and dermatitis acneiform for the RAM + ERL and PBO + ERL arms were similar between the ITT safety population and the exposure–safety population, as shown in Table 4. Exposure–safety relationship was first assessed by examining the observed incidences for the selected safety endpoints by ramucirumab exposure (*C*_min,*ss*_) quartiles. Although the observed incidences of each RAM + ERL *C*_min,*ss*_ quartile were greater than that of the PBO + ERL arm for all selected Grade ≥ 3 TEAEs and selected AESIs, there was no statistically nor clinically significant relationship between exposure and safety following ramucirumab 10 mg/kg Q2W dosing (Table 4; Online Resource 4). For any grade increased alanine aminotransferase (ALT) and aspartate aminotransferase (AST), a trend toward increasing incidences with increased exposure was observed, with the highest incidence observed in the Q4 group. Ordered categorical analysis were performed to evaluate the relationship between predicted measures of exposure and the incidences of any grade ALT or AST increased. Based on this analysis, there was no statistically significant relationship between ramucirumab exposure and increased incidence of ALT or AST (any grade or ≥ grade 3).

**Table 4:** RELAY Observed grade ≥ 3 TEAE and AESI incidence by quartile of ramucirumab *C*_min,*ss*_

	ITT safety population		Exposure–safety population					
	PBO + ERL	RAM + ERL	PBO + ERL	RAM + ERL^d^				
				*C*_min,*ss*_				
				Overall	RAM Q1	RAM Q2	RAM Q3	RAM Q4
	*N* = 225	*N* = 221	*N* = 225	*N* = 216	*N* = 54	*N* = 54	*N* = 54	*N* = 54
Ramucirumab conc (μg/mL)	–	–	–	10.1–208.0	10.1–74.9	75.1–89.6	89.8–108.0	109.0–208.0
*TEAE*								
Grade ≥ 3, *n* (%)								
Diarrhea^a^	3 (1)	16 (7)	3 (1)	16 (7)	4 (7)	4 (7)	3 (6)	5 (9)
Dermatitis acneiform^a^	20 (9)	33 (15)	20 (9)	33 (15)	10 (19)	6 (11)	7 (13)	10 (19)
*AESI*								
Any grade, *n* (%)								
Hypertension^a^	27 (12)	100 (45)	27 (12)	99 (46)	25 (46)	25 (46)	23 (43)	26 (48)
Proteinuria^b^	19 (8)	76 (34)	19 (8)	76 (35)	18 (33)	20 (37)	18 (33)	20 (37)
Liver failure/liver injury^a,c^								
ALT increased	70 (31)	94 (43)	70 (31)	93 (43)	21 (39)	27 (50)	17 (32)	28 (52)
AST increased	58 (26)	92 (42)	58 (26)	91 (42)	21 (39)	24 (44)	19 (35)	27 (50)
Grade ≥ 3, *n* (%)								
Hypertension	12 (5)	52 (24)	12 (5)	52 (24)	17 (32)	11 (20)	15 (28)	9 (17)
Proteinuria^b^	0 (0)	6 (3)	0 (0)	6 (3)	3 (6)	0 (0)	1 (2)	2 (4)

^a^Preferred term

^b^consolidated term

^c^analysis on grade ≥ 3 ALT and AST was not performed as there was < 2% difference in incidence between the ramucirumab and placebo groups

^d^Predicted *C*_min,*ss*_ exposure quartiles: RAM Q1 *C*_min,*ss*_ 10.1–74.9 μg/mL (< 25%); RAM Q2, *C*_min,*ss*_ 75.1–89.6 μg/mL (25–< 50%); RAM Q3, *C*_min,*ss*_ 89.8–108 μg/mL (50–< 75%); RAM Q4, *C*_min,*ss*_ 109–208 μg/mL (≥ 75%). *C*_min,*ss*_, minimum concentration at steady-state; *N*, number of patients, Q, quartile, RAM + ERL, ramucirumab plus erlotinib; PBO + ERL, placebo plus erlotinib; TEAE, treatment emergent adverse event, AESI, adverse events of special interest

Dose intensity and dose adjustments of ramucirumab and erlotinib were summarized by ramucirumab *C*_min,*ss*_ and *C*_min,1_ quartiles for the exposure–safety analysis population. The results were found to be generally consistent between these two exposure parameters. The relative dose intensity of ramucirumab or erlotinib was similar across the 4 exposure quartiles. There was no apparent relationship observed between ramucirumab exposure and dose adjustments of ramucirumab (dose delay, dose reduction, or dose omission). In addition, the percentage of patients with dose adjustments of ramucirumab in all 4 RAM + ERL quartiles was generally higher (76%) compared with the PBO + ERL arm (59%). There was also no apparent relationship observed between ramucirumab exposure and dose adjustments of erlotinib (dose reduction and dose omission). The percentage of patients with dose adjustments of erlotinib was generally similar between the PBO + ERL arm and all 4 RAM + ERL quartiles.

## Discussion

Monoclonal antibodies (mAbs) are used in the treatment of many diseases and have been shown to be effective anticancer agents. mAbs typically offer high target specificity and can possess considerable advantages over small-molecule drugs and conventional therapy, such as increased treatment efficacy and lower toxicity [14]. PK of mAbs is generally well understood. However, as several obstacles limit the diffusion of mAbs into tumor tissue, elucidating distribution and elimination mechanisms of systemically administered mAbs is challenging. Elevated interstitial fluid pressure in the tumor and high levels of target antigen in peripheral tumor tissue can limit tumor penetration. Consequently, the distribution of mAbs in tumor tissue is highly heterogeneous [14, 30].

mAbs are customarily characterized by low clearance and association with low volume of distribution, leading to a long half-life of up to several days. PopPK analysis and E–R analysis are tools utilized to evaluate the PK, safety, and effectiveness of mAbs. E–R analyses have become an instrumental part of clinical development and have helped improve dosing strategies in different tumor types [24, 31–33].

Findings from E–R analyses of ramucirumab 2L phase 3 studies investigating 8 mg/kg Q2W or 10 mg/kg Q3W suggested an opportunity to improve outcomes through modification of the dosing regimen. Data from these studies indicated that patients with lower ramucirumab exposure were responding less optimally in comparison to patients with higher exposure levels. Consequently, in RELAY, a ramucirumab 10 mg/kg Q2W regimen was utilized to increase drug concentration in serum across all patients through more frequent dosing regimen. We conducted an exploratory analysis on the patient data from RELAY to evaluate the E–R produced by the 10 mg/kg Q2W regimen. As predicted, ramucirumab steady-state concentration levels were higher with less inter-patient variability following 10 mg/kg Q2W compared with 10 mg/kg Q3W dosing used in REVEL. In addition, most patients in RELAY reached the target efficacious ramucirumab concentration of 50 µg/mL. The target exposure was also reached earlier in therapy with the more intense Q2W regimen compared with the Q3W regimen followed in REVEL. Moreover, it was hypothesized that the dosing regimen investigated in RELAY would not produce a large *C*_max_ increase relative to the approved dosing regimen from the 2L NSCLC indication (REVEL). The findings of this study confirmed this. This is an advantage as an increase in *C*_max_ may increase the potential safety risks.

Interestingly, ramucirumab pharmacokinetic parameter estimates, clearance, terminal half-life, and volume of distribution, were comparable to those in previous analysis of different indications [18, 19, 22]. In a PopPK meta-analysis by O’Brien et al. [22], body weight was identified as the only covariate with a clinically significant influence on the disposition of ramucirumab. Similarly, the patient population in RELAY was found to have no influence on the disposition of ramucirumab when compared with the populations, tumor types, and lines of therapy analyzed in other studies. Further, ramucirumab disposition was found to be similar in Asian and Caucasian patients.

RELAY met its primary endpoint of superior PFS for patients who received ramucirumab plus erlotinib versus placebo plus erlotinib, demonstrated in the primary analysis [13]. Analysis of PFS by *C*_min,1_ quartile demonstrated no significant relationship between the Kaplan–Meier PFS curves and ramucirumab exposure, indicating that there was no clear association between increased exposure and improved clinical outcome. A plausible explanation for the observed results is that the molecular target has been saturated with ramucirumab and the effects of the drug have been maximized. Comparable response to treatment was also observed in patients with the lowest exposure levels, suggesting that the dosing regimen produced effective serum concentrations across the patient population. Prior to the start of treatment in RELAY, baseline patient and disease characteristics generally did not indicate severe symptom burden or impaired quality of life among patients [34]. Consequently, this indicated a low level of cachexia in the population which prevented including the covariate in the analysis. Though a study by Turner et al. [35] advocates a relationship between mAb clearance and OS response in cancer as a consequence of cachexia, it is important to recall that the clearance of mAbs is strongly associated to the target expression (target mediated drug disposition). The level of target expression is differently impacted by the disease status, symptom burden, and cachexia burden depending on the target. Ramucirumab is not targeting the immune system and is not directly targeting tumor cells. Ramucirumab is acting on the vasculature of the tumor. In the context of the generally low disease burden (including cachexia) at treatment initiation for patients with *EGFR*-mutated NSCLC, there is a lower probability that ramucirumab clearance may be linked to the disease status in this patient population in the absence of significant change in body weight during study treatment.

In the RELAY trial, grade ≥ 3 TEAEs occurring in at least 5% of patients and at a > 2% higher incidence in the ramucirumab arm versus the control arm were hypertension, diarrhea, and dermatitis acneiform. Although the incidences of selected safety endpoints in each ramucirumab *C*_min,*ss*_ quartile were greater than that of the placebo plus erlotinib arm, there was no association between ramucirumab exposure and toxicity. Thus, no exposure–safety relationship was identified for the selected safety endpoints. Additionally, increased ramucirumab exposure did not appear to be associated with an increased percentage of dose adjustments for ramucirumab or erlotinib in the safety population over the range of exposures achieved by 10 mg/kg Q2W.

Though comparative analysis of REVEL and RELAY supports the Q2W regimen, the impact of the line of therapy on E–R must be considered. In oncology, additional determinants, notably prognostic factors, are reported to add complexity in characterizing E–R of mAbs [15]. The line of treatment and the duration of prior lines are important prognostic indicators as, in comparison to tumors receiving first-line treatment, tumors receiving second line treatment are likely more advanced, have a higher disease burden, and are harder to treat. Therefore, line of treatment may have an impact on E–R analyses. It is imperative to note that patients in REVEL received ramucirumab as second line treatment, whereas ramucirumab was administered as first-line treatment in the current study. A further disparity which may interfere with comparing results from each trial are the treatment combinations utilized. In REVEL, the effect of ramucirumab was investigated in combination with docetaxel, whereas RELAY examined the therapeutic effect of ramucirumab in combination with erlotinib. These factors may be contributing to the difference in E–R observed in the studies.

Although RELAY demonstrates the safety and efficacy of first-line combination treatment with ramucirumab 10 mg/kg Q2W plus erlotinib (150 mg/day) in *EGFR*-mutated metastatic NSCLC, this analysis is limited by several factors. The primary limitation was the use of a single dose level to identify E–R relationship. Analysis of further doses would increase the statistical power to establish an accurate relationship. However, this limitation was counter-balanced by comparatively analyzing the different dosing schedules investigated in REVEL and RELAY as well as incorporating data from the control arm. Though the ramucirumab PK data observed in RELAY are consistent with previously reported PK data following treatment with ramucirumab monotherapy and in combination with taxanes [18, 19, 22]. Furthermore, erlotinib PK data collected in both arms of RELAY showed similar erlotinib exposure, indicating negligible potential for PK drug–drug interaction between ramucirumab and erlotinib. Consequently, the results obtained from the ramucirumab PopPK model are robust and can be used for E–R analysis.

In conclusion, while it is necessary to confirm patient preference of therapeutic approach, these findings outline the clinical benefits of the RELAY regimen. The ramucirumab 10 mg/kg Q2W regimen led to a higher steady-state concentration and the target exposure was reached earlier in therapy compared with the previously explored regimen in the 2nd line setting. There was no indication of an E–R relationship, suggesting identification of an optimized dose of 10 mg/kg Q2W to improve outcomes across the patient cohort. The recommended 10 mg/kg Q2W ramucirumab dose combined with erlotinib (150 mg/day) is an efficacious and safe 1st line treatment for EGFR-mutated, metastatic NSCLC.

## Supplementary Information

Below is the link to the electronic supplementary material.

Supplementary file1 (DOCX 15 KB)

Supplementary file2 (DOCX 14 KB)

Supplementary file3 (DOCX 16 KB)

Supplementary file4 (DOCX 289 KB)

## Data Availability

Lilly provides access to all individual participant data collected during the trial, after anonymization, with the exception of pharmacokinetic or genetic data. Data are available to request 6 months after the indication studied has been approved in the US and EU and after primary publication acceptance, whichever is later. No expiration date of data requests is currently set once data are made available. Access is provided after a proposal has been approved by an independent review committee identified for this purpose and after receipt of a signed data sharing agreement. Data and documents, including the study protocol, statistical analysis plan, clinical study report, blank or annotated case report forms, will be provided in a secure data sharing environment. For details on submitting a request, see the instructions provided at www.vivli.org.
