# Supplementary material for: RELAY, ramucirumab plus erlotinib versus placebo plus erlotinib in untreated EGFR-mutated metastatic non-small cell lung cancer: exposure–response relationship
Source: Cancer Chemother Pharmacol. 2022 Jul 16;90(2):137–48. doi: 10.1007/s00280-022-04447-x (PMC9360106; doi:10.1007/s00280-022-04447-x)
Supplement: Supplementary file 1 — Supplementary file1 (DOCX 15 KB) [file 280_2022_4447_MOESM1_ESM.docx]

**RELAY, Ramucirumab plus Erlotinib versus Placebo plus Erlotinib in Untreated EGFR-Mutated Metastatic Non-Small Cell Lung Cancer: Exposure-Response Relationship**

Cancer Chemotherapy and Pharmacology

Kazuhiko Nakagawa^1^, Edward B. Garon, Ling Gao, Sophie Callies, Annamaria Zimmermann, Richard Walgren, Carla Visseren-Grul, Martin Reck

^1^Kindai University Faculty of Medicine, Osaka, Japan

**Correspondence to:**

Prof. Kazuhiko Nakagawa

Department of Medical Oncology, Kindai University, Faculty of Medicine, 377-2, Ohno-higashi, Osakasayama City, Osaka, 589-8511 Japan

Email: nakagawa@med.kindai.ac.jp

**Online Resource 1.** RELAY Ramucirumab predicted population pharmacokinetics for different regions.

|  | RAM+ERL | | | | |
| --- | --- | --- | --- | --- | --- |
|  | All Patients | | | | |
|  | All | East Asia | | | Other |
| PK Parameters  Geometric Mean (CV%) | N=216 | All N=161 | Japan N=104 | Non-Japan N=57 | N=55 |
| C_min,1_, μg/mL | 36.0 (32) | 35.5 (33) | 35.7 (37) | 35.1 (25) | 37.6 (26) |
| C_min,ss_, μg/mL | 88.3 (35) | 85.4 (35) | 85.4 (38) | 85.5 (28) | 97.1 (33) |

PK, pharmacokinetics; CV, coefficient of variation; C_min,1_, minimum concentration after first dose; C_min,ss_, minimum concentration at steady-state; NSCLC, non-small cell lung cancer; N, number; RAM+ERL- ramucirumab plus erlotinib.
