# Supplementary material for: RELAY, ramucirumab plus erlotinib versus placebo plus erlotinib in untreated EGFR-mutated metastatic non-small cell lung cancer: exposure–response relationship
Source: Cancer Chemother Pharmacol. 2022 Jul 16;90(2):137–48. doi: 10.1007/s00280-022-04447-x (PMC9360106; doi:10.1007/s00280-022-04447-x)
Supplement: Supplementary file 2 — Supplementary file2 (DOCX 14 KB) [file 280_2022_4447_MOESM2_ESM.docx]

**RELAY, Ramucirumab plus Erlotinib versus Placebo plus Erlotinib in Untreated EGFR-Mutated Metastatic Non-Small Cell Lung Cancer: Exposure-Response Relationship**

Cancer Chemotherapy and Pharmacology

Kazuhiko Nakagawa^1^, Edward B. Garon, Ling Gao, Sophie Callies, Annamaria Zimmermann, Richard Walgren, Carla Visseren-Grul, Martin Reck

^1^Kindai University Faculty of Medicine, Osaka, Japan

**Correspondence to:**

Prof. Kazuhiko Nakagawa

Department of Medical Oncology, Kindai University, Faculty of Medicine, 377-2, Ohno-higashi, Osakasayama City, Osaka, 589-8511 Japan

Email: [nakagawa@med.kindai.ac.jp](mailto:nakagawa@med.kindai.ac.jp)

**Online Resource 2.** RELAY Summary of erlotinib noncompartmental pharmacokinetic parameters in the ramucirumab plus erlotinib and the placebo plus erlotinib treatment arms.

|  | **Treatment arm** | |
| --- | --- | --- |
| **Parameter** | **RAM+ERL (n=11)** | **PBO+ERL (n=15)** |
| C_max_ , ng/mL  Mean (CV%) | 2520 (20) | 2210 (27) |
| T_max_ , hr  Median (min-max) | 2.1 (0.75-7.17) | 2.0 (0.97-5.87) |
| AUC_24_ , ng⋅hr/mL  Mean (CV%) | 41,200 (20) | 33,400 (34)* |

AUC_24_, area under plasma concentration-time curve over 24 hours; Cmax, maximum observed drug concentration; CV, coefficient of variation; n, number; T_max_, time of C_max_;

* Data not available for two patients.
