# Supplementary material for: RELAY, ramucirumab plus erlotinib versus placebo plus erlotinib in untreated EGFR-mutated metastatic non-small cell lung cancer: exposure–response relationship
Source: Cancer Chemother Pharmacol. 2022 Jul 16;90(2):137–48. doi: 10.1007/s00280-022-04447-x (PMC9360106; doi:10.1007/s00280-022-04447-x)
Supplement: Supplementary file 3 — Supplementary file3 (DOCX 16 KB) [file 280_2022_4447_MOESM3_ESM.docx]

**RELAY, Ramucirumab plus Erlotinib versus Placebo plus Erlotinib in Untreated EGFR-Mutated Metastatic Non-Small Cell Lung Cancer: Exposure-Response Relationship**

Cancer Chemotherapy and Pharmacology

Kazuhiko Nakagawa^1^, Edward B. Garon, Ling Gao, Sophie Callies, Annamaria Zimmermann, Richard Walgren, Carla Visseren-Grul, Martin Reck

^1^Kindai University Faculty of Medicine, Osaka, Japan

**Correspondence to:**

Prof. Kazuhiko Nakagawa

Department of Medical Oncology, Kindai University, Faculty of Medicine, 377-2, Ohno-higashi, Osakasayama City, Osaka, 589-8511 Japan

Email: [nakagawa@med.kindai.ac.jp](mailto:nakagawa@med.kindai.ac.jp)

**Online Resource 3.** RELAY Baseline Factors Before and After Matching, by **C_min,1_** Quartiles.

| **Baseline Factors** | **Before Matching** | | | **After Matching** | | |
| --- | --- | --- | --- | --- | --- | --- |
|  | **RAM+ERL** | **PBO+ERL** | **P-value** | **RAM+ERL** | **PBO+ERL** | **P-value** |
| Q1, N | 54 | 225 |  | 54 | 54 |  |
| Age (<65 vs. ≥65) | 0.46 | 0.51 | 0.6498 | 0.46 | 0.46 | 1.0000 |
| ECOG PS (0 vs. ≥1) | 0.43 | 0.53 | 0.2251 | 0.43 | 0.43 | 1.0000 |
| Q2, N | 54 | 225 |  | 54 | 54 |  |
| Age (<65 vs. ≥65) | 0.50 | 0.51 | 1.0000 | 0.50 | 0.50 | 1.0000 |
| ECOG PS (0 vs. ≥1) | 0.50 | 0.53 | 0.7624 | 0.50 | 0.50 | 1.0000 |
| Q3, N | 54 | 225 |  | 54 | 54 |  |
| Age (<65 vs. ≥65) | 0.44 | 0.51 | 0.4506 | 0.44 | 0.44 | 1.0000 |
| ECOG PS (0 vs. ≥1) | 0.57 | 0.53 | 0.6488 | 0.57 | 0.57 | 1.0000 |
| Q4, N | 54 | 225 |  | 54 | 54 |  |
| Age (<65 vs. ≥65) | 0.46 | 0.51 | 0.6498 | 0.46 | 0.46 | 1.0000 |
| ECOG PS (0 vs. ≥1) | 0.56 | 0.53 | 0.7629 | 0.56 | 0.56 | 1.0000 |

ECOG PS, Eastern Cooperative Oncology Group performance status; RAM+ERL- ramucirumab plus erlotinib; PBO+ERL- placebo plus erlotinib; N, number
