# Supplementary material for: RELAY, ramucirumab plus erlotinib versus placebo plus erlotinib in untreated EGFR-mutated metastatic non-small cell lung cancer: exposure–response relationship
Source: Cancer Chemother Pharmacol. 2022 Jul 16;90(2):137–48. doi: 10.1007/s00280-022-04447-x (PMC9360106; doi:10.1007/s00280-022-04447-x)
Supplement: Supplementary file 4 — Supplementary file4 (DOCX 289 KB) [file 280_2022_4447_MOESM4_ESM.docx]

**RELAY, Ramucirumab plus Erlotinib versus Placebo plus Erlotinib in Untreated EGFR-Mutated Metastatic Non-Small Cell Lung Cancer: Exposure-Response Relationship**

Cancer Chemotherapy and Pharmacology

Kazuhiko Nakagawa^1^, Edward B. Garon, Ling Gao, Sophie Callies, Annamaria Zimmermann, Richard Walgren, Carla Visseren-Grul, Martin Reck

^1^Kindai University Faculty of Medicine, Osaka, Japan

**Correspondence to:**

Prof. Kazuhiko Nakagawa

Department of Medical Oncology, Kindai University, Faculty of Medicine, 377-2, Ohno-higashi, Osakasayama City, Osaka, 589-8511 Japan

Email: nakagawa@med.kindai.ac.jp


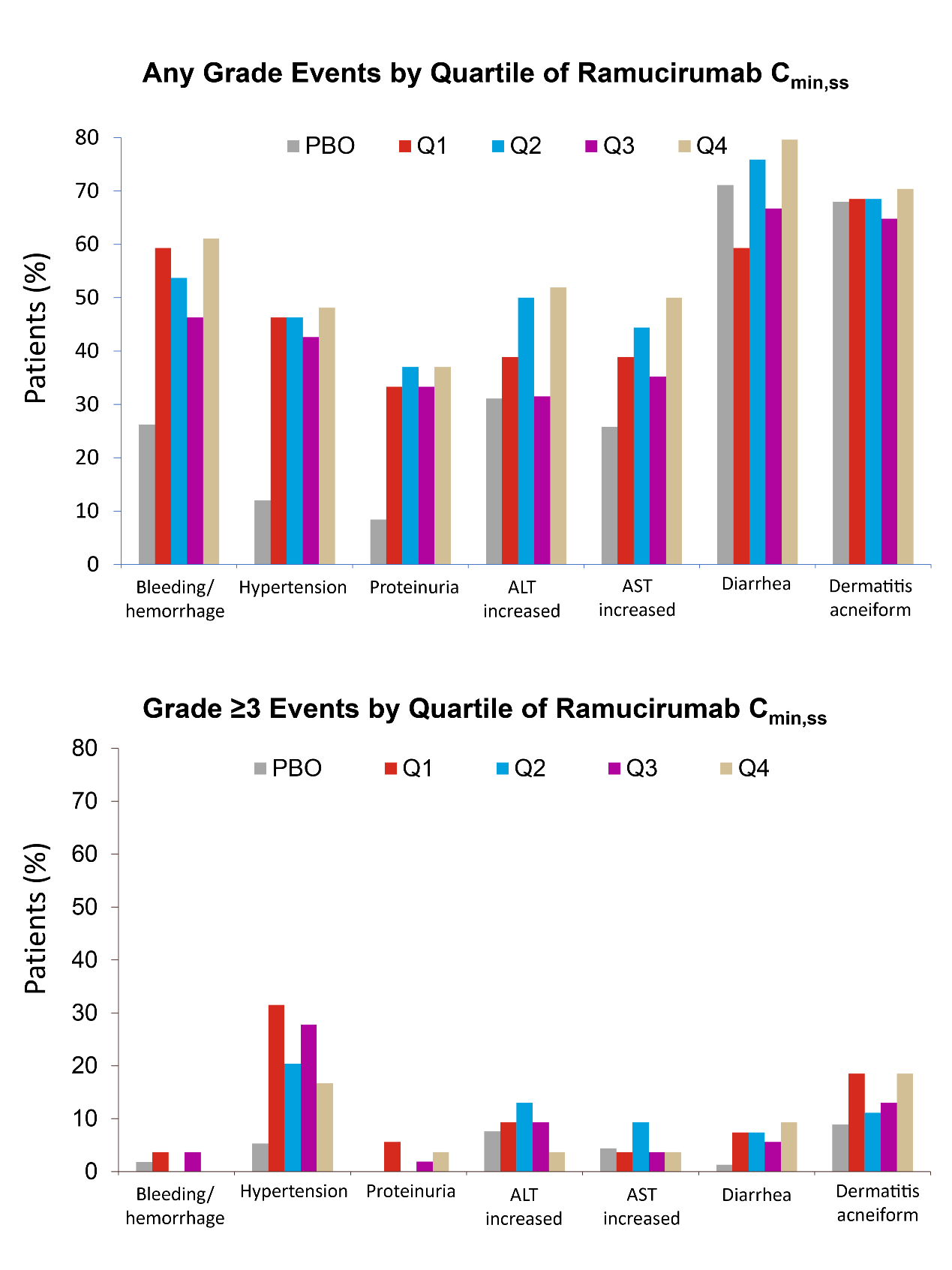


**Online Resource 4.** RELAY Observed grade ≥3 TEAE and AESI incidence by C_min,ss_ quartile of ramucirumab. Predicted C_min,ss_ exposure quartiles: RAM Q1 C_min,ss_ 10.1 – 74.9 μg/mL (<25%); RAM Q2, C_min,ss_ 75.1 – 89.6 μg/mL (25 - <50%); RAM Q3, C_min,ss_ 89.8 – 108 μg/mL (50 - <75%); RAM Q4, C_min,ss_ 109 – 208 μg/mL (≥75%). C_min,ss_, minimum concentration at steady-state; PBO- placebo; Q, quartile; RAM- ramucirumab
